# Virtual Patients in a Behavioral Medicine Massive Open Online Course (MOOC): A Qualitative and Quantitative Analysis of Participants’ Perceptions

**DOI:** 10.1007/s40596-017-0706-4

**Published:** 2017-04-07

**Authors:** Anne H. Berman, Gabriele Biguet, Natalia Stathakarou, Beata Westin-Hägglöf, Kerstin Jeding, Cormac McGrath, Nabil Zary, Andrzej A. Kononowicz

**Affiliations:** 1 Karolinska Institutet, Center for Psychiatry Research, Stockholm, Sweden; 2 Stockholm University, Stockholm, Sweden; 3 The Stress Research Clinic, Stockholm, Sweden; 4 Jagiellonian University Medical College, Krakow, Poland

**Keywords:** MOOC, Behavioral medicine, Case-based learning, Virtual patient, Evaluation, Qualitative content analysis

## Abstract

**Objective:**

The purpose of this article is to explore learners’ perceptions of using virtual patients in a behavioral medicine Massive Open Online Course (MOOCs) and thereby describe innovative ways of disseminating knowledge in health-related areas.

**Methods:**

A 5-week MOOC on behavioral medicine was hosted on the edX platform. The authors developed two branched virtual patients consisting of video recordings of a live standardized patient, with multiple clinical decision points and narration unfolding depending on learners’ choices. Students interacted with the virtual patients to treat stress and sleep problems. Answers to the exit survey and participant comments from the discussion forum were analyzed qualitatively and quantitatively.

**Results:**

In total, 19,236 participants enrolled in the MOOC, out of which 740 received the final certificate. The virtual patients were completed by 2317 and 1640 participants respectively. Among survey respondents (*n* = 442), 83.1% agreed that the virtual patient exercise was helpful. The qualitative analysis resulted in themes covering what it was like to work with the virtual patient, with subthemes on learner-centered education, emotions/eustress, game comparisons, what the participants learned, what surprised them, how confident participants felt about applying interventions in practice, suggestions for improvement, and previous experiences of virtual patients.

**Conclusions:**

Students were enthusiastic about interacting with the virtual patients as a means to apply new knowledge about behavioral medicine interventions. The most common suggestion was to incorporate more interactive cases with various levels of complexity. Further research should include patient outcomes and focus on interprofessional aspects of learning with virtual patients in a MOOC.

Massive online open courses (MOOCs) have recently become widely available on numerous topics through university-sponsored online platforms. Large volumes of students, usually numbered in the thousands and up to 200,000 for the most popular topics, enroll in MOOCs, although the students who persevere all the way to obtaining final certificates constitute a small percentage of original enrollees [1]. Still, the large quantity of students achieving final certificates translates into dissemination of academic knowledge to larger groups than ever before, far beyond the capacity of traditional classroom-based education. MOOCs in the health sciences offer huge potential for disseminating scientifically verified knowledge on human health, with unknown possibilities for directly impacting human health. Using standardized patients is particularly efficient in behavior change counseling curricula [2], but is impossible at a massive scale in distance education. We were therefore looking for a high-quality alternative by offering virtual patients to MOOC students, an option which has been previously demonstrated to be of equivalent effectiveness to standardized patients, albeit in a different context [3].

Virtual patients are defined as “interactive computer simulation of real-life clinical scenarios for the purpose of healthcare and medical training, education or assessment” [4]. Over the past decade or so, virtual patients have increasingly been used within health professional education to help students prepare for working with real patients [5]. Research has shown that using virtual patients in health professional educational instruction is significantly better than no such intervention at all, for teaching clinical reasoning, knowledge outcomes, and other skills; virtual patients also have small effects in comparison to noncomputer-based cases [6–8]. In choosing from among the variety of virtual patient types available [9], we decided to use branched narrative interactive patient scenarios as a highly accessible, time, and cost-effective method to teach clinical reasoning using simple multimedia (audio, video, and interactive elements) presented online [10].

Despite the huge volume of MOOCs produced over the past few years, virtual patients have not, to our knowledge, been used yet within MOOCs. We integrated two interactive patient scenarios on stress and sleep problems, respectively: in KIBEHMEDx, a MOOC on the edX platform that ran live over 5 weeks in the autumn of 2014, as a self-paced 5-month course in the spring of 2016 and, in the fall of 2016, in a re-branded live 6-week version including a new section on innovative delivery of behavioral medicine health services. In between live versions of the course, it is archived with all course material openly available [11, 12]. This article presents a quantitative and qualitative analysis of MOOC participants’ experiences of interacting with the virtual patients based on the online MOOC discussion forum and the final course exit survey in the first live course in 2014. Our overriding aim was to explore innovative routes of dissemination of behavioral medicine knowledge, with potential implications for the future development of health-related MOOCs incorporating virtual patients.

## Methods

The KIBEHMEDx course contains five main sections detailed in Table 1. For technical information about the course and the virtual patients, see [11].

**Table 1:** Structure and content of the KIBEHMEDx MOOC launched in September 2014

Section	Description
1	Section 1 covers Health Behaviors and Motivation to Change, where basic tools and processes of Motivational Interviewing (MI) are taught [33]
2	Section 2 covers Stress and Coping, beginning with an introduction to stress that covers basic concepts of stress and disease and the physiology of stress mechanisms and continuing with physical and mental effects of stress, specifically musculoskeletal disorders, mental health and exhaustion syndromes. At the end of Section 2, interventions for exhaustion syndromes are presented, including functional behavior analysis, control of internal and external stressors, mindfulness, and exercises inspired by acceptance and commitment therapy (ACT), focusing on values and goals as well as defusion [34]. The first virtual patient interactive scenario on stress-related problems is the final part of this section
3	Section 3 covers Sleep, beginning with basic scientific concepts in sleep research and continuing with diagnosis and treatment of sleep disorders, followed by techniques for facilitating sleep, such as supporting circadian rhythms, balancing sleep and waking times, sleep hygiene, stimulus control, relaxation training, and handling distressing thoughts and emotions. The second virtual patient interactive scenario on sleep-related problems ends this section
4^a^	Section 4 covers Physical activity, encompassing physical activity and health, promotion of physical activity, and physical activity prescription within the health care context
5^b^	Section 5 focuses on Everyday behaviors and interpersonal communicable disease control, including hand hygiene, responsible sexual behavior, and responsible alcohol use. At the end of this section, the final course assignment is presented, involving a final project where students describe a process of behavior change in a live patient, continued change in one of the virtual patient scenarios, in a friend or family member, or in themselves

^a^Now Section 5 following the addition of a new section on innovative delivery of behavioral medicine health services in late autumn 2016

^b^Now Section 6

**Fig. 1:**
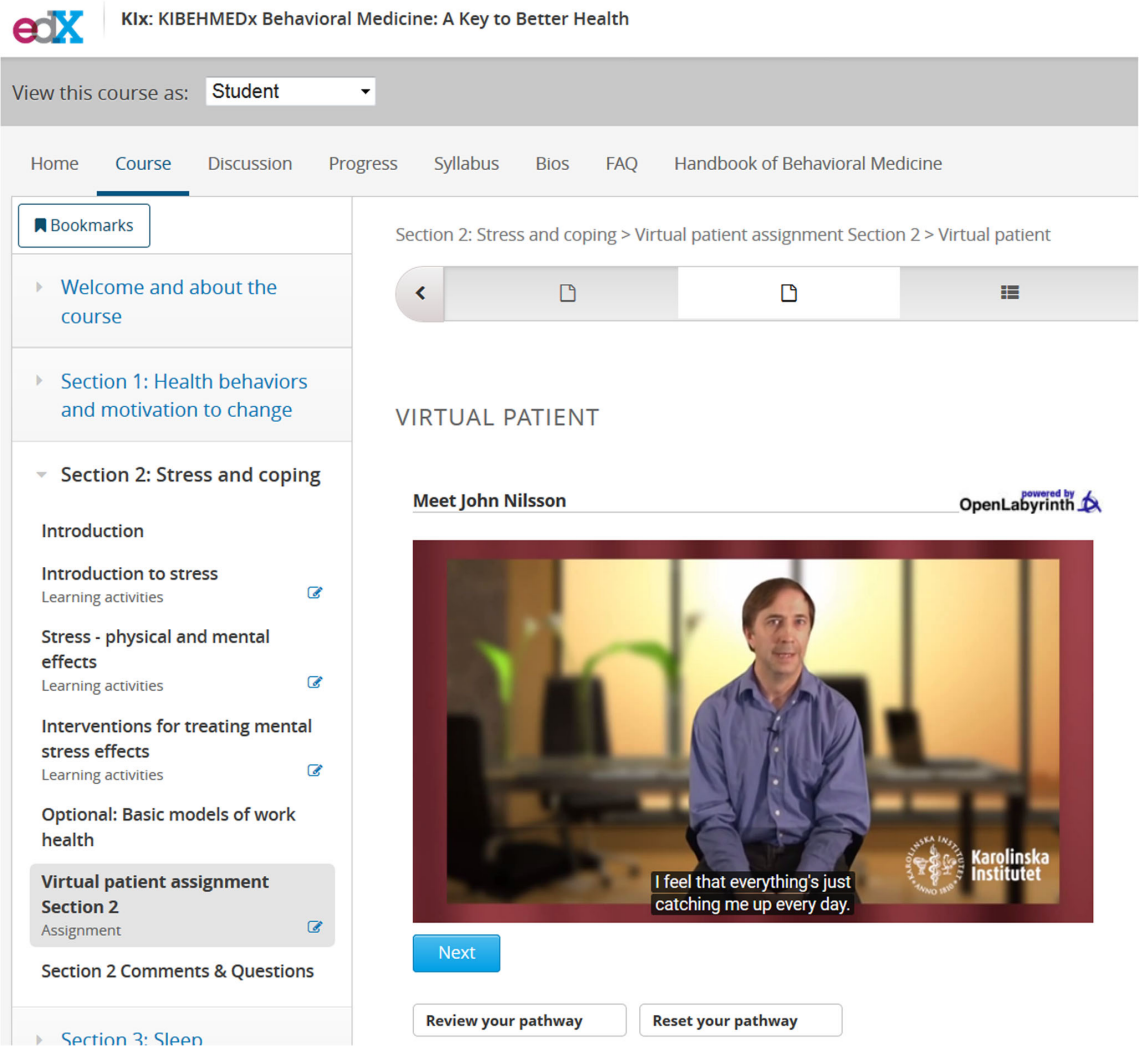
The virtual patient presented in KIBEHMEDx, the edX Behavioral Medicine course

The content for the virtual patients was developed as follows. The authors (AHB, GB, KJ, NS, AAK) used the 12 specific guidelines (Tips) for creating virtual patients, provided by Posel and colleagues [13] in order to construct the two virtual patient scenarios used in KIBEHMEDx. The first virtual patient focused on stress-related problems and was presented in the second section of the MOOC. The second virtual patient case covered sleep-related topics and was presented in the following third section. We selected branched narrative as the design model, meaning that learners select the best available course of action from predefined options to decide how the case will unfold [10]. This allows for high learner interactivity, an experience we wanted to prioritize given recent criticism of MOOCs as unsophisticated forms of passive learning through short video lectures [14]. We structured the scenarios around John Nilsson, a high school teacher pressured by increased administration at work and family life with small children and a wife longing for a third child (see Fig. 1).

**Fig. 2:**
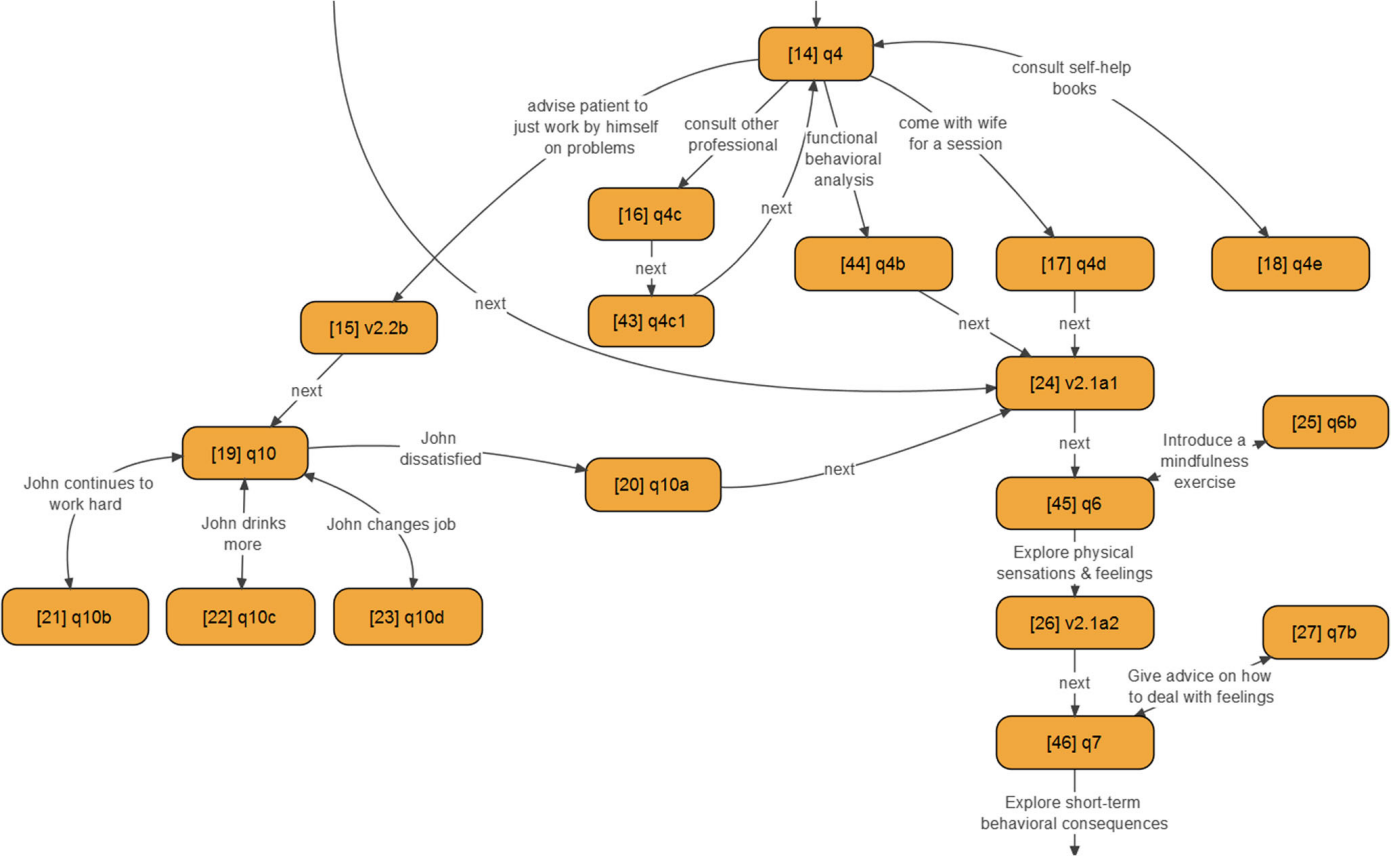
An overview of the number of options available to students at each decision point in the virtual patient exercise; the *numbers* indicate unique identifiers of the decision nodes

The purpose of the two scenarios was to teach the students skills of engaging with the patient via attention to verbal and body language, as well as when to apply intervention skills. The interventions were taught in the earlier parts of the stress and sleep sections respectively, which preceded the virtual patient exercise in each section. We made the case more challenging and complex by offering alternative scenarios depending on the learner choices. See Fig. 2 for an example of the type and number of options available to students at decision points in the case.

We hired a professional actor and film team and involved clinicians to shoot the short role-played video clips for the case. Also, we matched branches to case objectives, where learners were able to select interventions taught in the course, for example functional behavioral analysis, and view the clinician’s work with the patient on this task. Assessment was also used in the instructional path, both multiple choice questions on the content, as well as free text questions and posts shared in the discussion forum. In addition, the participants were given the opportunity to learn from feedback given while exploring the pathways regarded as suboptimal based on the course content.

We chose the freely available Open Labyrinth application [15] to program the virtual patient scenarios [16, 17]. This application supports online authoring and updating of cases, export of activity logs, and the possibility of exchanging cases to be used in other settings. We supported learners by linking the virtual patient to course material when necessary, for example to remind them of the content of the mindfulness exercise in the stress scenario. Learners were able to see the outcomes of their choices, either in text-based descriptions or in video clips. The stress-related virtual patient included 80 screen cards (i.e., distinct stages in the interaction with the virtual patient) with 18 branching points (at each such point, it was possible to select one of several intervention option). The sleep-related virtual patient was slightly shorter with 61 screen cards and 14 branching points. The total authoring time of two interactive patient scenarios including planning and writing the content, recording, negotiating technical issues, testing, and revision involved around 150 h work altogether.

### Measures

MOOC participants reported their experiences of working with a virtual patient through two channels: the instructor-moderated online discussion forum within Sections 2 and 3 of KIBEHMEDx and an online exit survey following the end of the live version of KIBEHMEDx. Four questions were asked at the end of each virtual patient assignment for Sections 2 and 3, and participants posted their responses on the general discussion forum. Three items from the 33 question exit survey, sent out to all course participants after the deadline for submission of the final course project assignment (October 24, 2014), concerned participant experience with the virtual patient scenarios. The questions from the discussion forum and exit survey are available from the corresponding author on request.

### Participants

Initially, 19,236 students were enrolled in KIBEHMEDx, representing 185 countries with the largest groups from the USA (27.6%), India (8.8%), and the UK (4.8%). Students who actually logged in on the course website during Section 1 numbered 4586 (23.8%); completion of the stress-related virtual patient assignment was declared by 2317 (50.5%) of Section 1 participants (12.1% of total enrollees), and completion of the sleep-related virtual patient assignment was declared by 1640 (35.8%) of Section 1 participants (8.5% of total enrollees). Of the users who declared completing at least one of the virtual patients and indicated their gender, 856 (55.6%) were women. Participants’ median age was 37 years. Regarding educational background, 16.3% stated they had high school diploma or less, 35.0% had a college degree, while 46.1% stated they had a graduate degree. Participants who completed the assignment spent an average of 48 min on the stress-related virtual patient and 39 min on the sleep-related virtual patient. Most of the participants who completed the second virtual patient had previous experience with the first virtual patient assignment, with only 60 new users. A total of 740 participants obtained honor certificates in the course, indicating that they had completed 65% of required course materials (16.1% of Section 1 participants; 3.8% of original enrollees).

The exit survey sent to all MOOC participants resulted in responses from 479 course participants by November 4th, 2014. Among survey respondents, 236 (49.3%) were practicing health professionals or students in the health professions, 189 (39.5%) were participants from other professional groups, and the remaining 11.3% selected “other” as answer to the question on professional background.

Regarding ethical issues, the research reported here falls outside of the vetting process of research in accordance with the Swedish Ethical Review Act (2003:460) because it does not handle sensitive personal data as defined in the Swedish Personal Data Act, §13 (nor is it invasive or includes biological samples from human subjects). Nonetheless, according to edX praxis in compliance with ethical guidelines, at registration, all course participants gave their informed consent that their comments in the discussion forum and responses to the exit survey could be used, in unidentified form, in coming research publications.

### Quantitative and Qualitative Analysis

One question from the exit survey was analyzed quantitatively with descriptive statistics. All text-based comments concerning the learner experience of interacting with the virtual patient scenarios were analyzed using qualitative content analysis, a systematic method of analysis which can be used to organize text-based data in order to extract units of meaning and sort them into categories, themes, or patterns [18]. For the present paper, we (AHB, BWH) began by formulating central themes deductively based on the questions asked in the discussion forum and the exit survey. In the second stage, we coded meaning units and defined and re-defined categories and subcategories through an inductive, data-driven process of reading and re-reading the material. A quantitative analysis of the frequency of comments per code was also performed. The comments were culled from the discussion forums for sections 2 and 3 and from the exit survey responses (*n* = 1375). In the third stage, the data were reviewed after new codes were added so that comments could be categorized anew, where appropriate. In a fourth stage, a secondary inductive analysis of the qualitative material in each theme was undertaken to identify additional content-based subthemes (AAK). The results encompass findings from all four stages of analysis.

## Results

### Quantitative Exit Survey Analysis

One exit survey question (Q30) concerned whether the participants agreed that the virtual patients were a helpful exercise. Of the 479 respondents, 442 (92.3%) answered this question, with 280 (58.5%) strongly agreeing, 118 (24.6%) agreeing, 39 (8.1%) neither agreeing nor disagreeing, 4 disagreeing (0.8%), and 1 person strongly disagreeing (0.2%). This amounted to 90.0% (398 of 442) positive responses, confirming that the virtual patients were a helpful exercise.

### Qualitative Discussion Forum and Exit Survey Analysis

A total of 1375 comments were collected from the discussion forum and the free text responses to the exit survey questions. Seven general themes, building on the questions from the discussion forum and exit survey, were formulated as a basis for the first coding of the material. In the second coding stage, categories were formulated for further analysis; themes 1, 2, and 3 also generated subcategories. A list of the themes and categories, with examples of comments as well as quote frequencies within the total number of comments and within each theme/category, is available from the corresponding author on request.

Working with the virtual patient was generally experienced positively, and a total count of words with positive valence generated 464 items [word stems practic-e/al (32.3%, *n* = 150), interest-ing/ed (22.8%, *n* = 106), and enjoy-ed/able (13.1%, *n* = 61), as well as the words good (22.6%, *n* = 105) and useful (9.1%, *n* = 42)]. The general contents of each theme and subtheme are summarized below.

1. What It Was Like to Work with the Virtual Patient

Participants generally found the virtual patient exercise interesting and enjoyed working with his case, finding this useful training for treating real patients. Theme 1 was the largest with 46.0% of all comments, with 89.6% of these falling in the “positive opinions” category. Respondents appear to have appreciated the chance to experiment with different possible responses to the virtual patient, enabling them to see varying results and reactions on the part of the patient. The feedback and explanations as to why some alternatives might not be beneficial for the patient were also an appreciated feature. John Nilsson, as virtual patient, is complimented as appearing realistic, with many commentators identifying with the virtual patient’s problems and reflecting about the virtual patient as if he were a real person. The exercise is also praised for being good practice in treating patients without the risk of harming a real person, as well as giving the participants more confidence in treating patients by practicing on a virtual patient. Several participants also wished to have even more virtual patient learning experiences.

The secondary analysis generated subthemes of *learner-centered education*, *emotions/eustress*, and *game comparisons.* For the first subtheme, participants emphasized their experience of the virtual patients as a tool that empowers their autonomy by providing an opportunity to shape their learning process individually:

*I really like the concept of the interaction with the virtual patient because you really create the scenario yourself…. I liked exploring the different options and it gives me a lot of ideas to deal with my patients.*

*Firstly I was surprised at how well the exercise is made technically…[as] it actually did give me a sense of control over the discussion.*

For the second subtheme, we observed signs of an emotional approach to the virtual patient task when choices on selecting the next steps caused tension, which was later soothed by positive and constructive feedback after the decision was made:

*I was nervous at first - wanting to do ‘the right thing’ but my responses were sympathetically handled so I didn’t feel too stressed.*

*Working with John required attention and [I] noticed that my heart rate [increased] and [that I had] some feelings of tension. It was actually intense as I wanted John to interact [with me] and become interested in solving his problems.*

Critical opinions regarding interactions with the virtual patient (6.1% of Theme 1) included hesitation concerning the approach of listening more and talking less, which was reflected on as troublesome if patients require many questions and encouragement from the counselor in order to be vocal at all. Some criticized the virtual patient for being unrealistic compared to real-life patients, who might be more unpredictable and less open to change and accepting treatment compared to the virtual patient. Another criticism concerned the need to have a functioning Internet connection in order to participate in the exercise.

For the third subtheme, some participants identified similarities between virtual patients and games, where for some this had positive effect on their perception of the exercise, whereas others rejected it for the same reason:

*Working with a virtual patient is phenomenal. It reminds me of… [computer] game[s]and [makes me aware] that… a lot of the energy [was] consumed in just creating this course”*

*“But the second time we worked with VP [virtual patient] - I was not so enthusiastic, because I perceived it more like a game, not like real responsible work.*

Theme 2: What Participants Learned from the Interactions

Participants expressed themselves in two categories, with about three-fourths of the comments concerning “concrete interventions learned,” such as when participants mention having learned Motivational Interviewing (MI), and about a fourth referring to “personal lessons learned,” where participants express more general realizations about themselves, for example being a good listener. Many of the participants point to the realization that they need to listen more patiently and talk less when treating a patient, without rushing to give advice and solutions, and that they need to formulate questions carefully in order to give patients a good chance to express themselves, for example using open questions. Participants also experienced a need to pay attention to details such as body language when interacting with a patient. A recurring realization is that the patient needs to be motivated and come up with his or her own suggestions of how to improve their well-being, and that the counselor needs to adapt her style rather than try to force change in the patient. Altogether, participants appeared appreciative about learning MI as a technique, and the positive and empathic counselor was perceived as inspirational. Many comments also concerned more general personal realizations and newfound knowledge. Several participants mentioned having learned to focus on one issue at a time when treating patients with various problems, and that they need to have patience because it can take some time before treatment is successful. The exercise also appears to have been useful for participants in setting an example of how to balance listening and intervening at appropriate times as a counselor.

The secondary analyses generated the subtheme *learning from errors*, where we noted in several comments that participants were aware of the opportunity to learn from their errors in the virtual patient activity, and actually took advantage of this experience. This is best recognizable in learners who reported they had deliberately explored wrong paths of action to learn from the corrective and consequential feedback embedded in our virtual patients.

*First I [chose how] I thought I would react in conversation with John, then I tried all other alternatives to see what they [would] lead to. I’ve learnt a lot from this process*

*“What I liked was when I selected an answer that was not the optimum choice, it was explained why that choice might not have been the best for that situation and I learned from that.*

Theme 3: What Surprised the Participants

Participant responses often centered on either unexpected behavior from the patient or surprise about the virtual patient as a learning tool, thus generating the categories “surprises about the virtual patient” (comprising over half the comments in this theme) and “surprises regarding learning aspects of the course” (28.8% of theme comments). Some participants describe their surprise at how the virtual patient opened up rather quickly. Participants also perceived the virtual patient as surprisingly real. Some participants describe being surprised by the virtual patient’s commitment to the treatment he was suggested to try, while others were generally surprised by the methods taught, particularly when treating sleep problems.

The secondary analysis generated the subtheme of *innovation in comparison to other types of learning*, where many participants indicated that the virtual patients were a new experience which they compared to methods used previously in their learning (e.g., chalk-board lectures, multiple-choice tests or passive video clips). The generally expressed that they preferred the new development.

*Having been brought up in the classroom, teacher/lecturer, chalk board era I found it very enlightening to learn this way.*

*I was surprised at how seeing an actor, or someone pretending to be in a situation, [was more] … helpful [for my learning] compared to just a question and answer type of test*

Theme 4: Participant Confidence in Using Interventions Taught

Many participants seemed comfortable and inspired to use the methods learned through the virtual patient exercise in real life with family, friends, for themselves, and also with clients or patients. Several participants reflected over their own behavior when interacting with others and those who claimed to be good listeners seemed to feel affirmed by the virtual exercise while others expressed a desire to become better listeners. Some participants stated that real-life scenarios do not include the convenience of being offered suggestions when treating patients as in the virtual patient exercise, making them feel unsure about how to treat patients completely on their own. Overall, participants indicated their appreciation of the structured step-by-step approach and wanted to incorporate it in interaction with others. Several participants said they had learned a lot but still needed more practice in order to feel confident.

Theme 5: Suggestions for Improvements

About 5% of all comments concerned suggestions for improvements, coded in 7 categories, with the largest being “requests for more multiple choice options and for more information” (about a third of the theme comments) and one in five comments being uncategorizable. Most of the remaining comments concerned adjustments in virtual patient videos: making them shorter, more numerous, more challenging, or closer to real life. Three participants suggested that the virtual patient scenario be made “downloadable.” The “uncategorized requests” included suggestions from criticizing the interior decoration behind the virtual patient to more content-oriented suggestions such as including virtual patients with more varied backgrounds and problems. An additional suggestion was to add an option to save the virtual patient session to allow resuming it on another occasion and to display the whole branching model underlying the virtual patient upon resolution of the case. Many exit survey respondents to Q30 and Q33 had no suggestions for improvement. Among the 39 who neither agreed nor disagreed that the virtual patient was a helpful exercise, few gave any suggestions on how to improve the learning experience, and the four who disagreed that the virtual patient was helpful had suggestions like having the video actors speak more loudly or improving the quality of the recording. The one respondent who strongly disagreed that the virtual patient was a helpful exercise did not give any suggestions on improvement.

Theme 6: Previous Participant Experiences Working with Virtual Patients

Sixteen participants had previously experienced virtual patients. Some participants who had previously worked with virtual patients described the KIBEHMEDx virtual patient as more rewarding because they considered the exercise more realistic compared to their earlier encounters with virtual patients. A summary of the responses to the exit survey question about previous experiences with virtual patients is that the experience of working with a virtual patient was generally perceived as an effective learning method.

Theme 7: Other Comments about the Virtual Patient

About a fifth of all the comments were in Theme 7, covering “other” content that fell outside specific experience with KIBEHMEDx, with two-thirds of these comments categorized as “not applicable or irrelevant.” Altogether, it seemed that, in this theme, some participants lost their focus on commenting on the exercise as a learning method and rather expressed opinions about the virtual patient as a person or about themselves.

## Discussion

Content development of the virtual patient in the KIBEHMEDx MOOC followed 12 tips on developing case content compiled based on existing literature [13]. The two branched virtual patient scenarios on stress- and sleep-related problems were designed to be relevant, realistic, engaging, challenging, and instructional [19], and indeed, 90% of the course participants who responded to the course exit survey agreed or strongly agreed that the exercise was helpful to their learning. The qualitative analysis of participant comments generated seven themes, with the overall trend being that the experience was interesting, enjoyable, and a good learning tool for applying theoretical knowledge in a practical format.

Virtual patients in the MOOC gave the participants an opportunity to shape their own learning by selecting different branches in the narration and taking part in related inter-professional discussions. This is a step towards learner-centered education, which is widely advocated for medical education [20]. Participants were astonished that the virtual patient seemed so realistic, and some were surprised by the nature of the interventions taught. We interpret the observed emotional reactions to the content as a positive sign of motivation that is likely to leave a memorable learning experience. Also, two out of three participants felt confident in applying the behavioral methods taught with others or in their own lives, in comparison to less than one in five who did not feel confident in applying the techniques. Some participants had previous experience of working with a virtual patient, and their general view was that this virtual patient was more realistic than their previous experiences. Game-informed elements of virtual patients [21] noticed in our course by some of the participants were both welcomed and rejected. We interpret that as a sign of the need for personalized learning, leaving the student a choice of the form of learning content. This might not be affordable in courses on small scale, but is possible in MOOCs. As the virtual patient designers, we were pleased by the extent of the positive responses (especially referring to the authenticity of the experience), given the relatively simple technical challenges involved in offering interactive patient scenarios. The importance of body language in the virtual patient activity was also stressed by several of the MOOC participants. We are aware that much of the appraisal given was in fact addressed to the video-recorded human actor who role-played the virtual patient. It remains an interesting research question to what degree this effect could be repeated with a computer-generated virtual character [22]. Virtual characters, as elements of user interface, are applied in a growing number of e-learning tools as being more flexible, but are so far of limited expressiveness and are not preferred by students [22]. Requests for improvements varied, but the most common was to have more exercises with virtual patients and to create virtual patients with more varied backgrounds and problems. From an interprofessional perspective, adding virtual patient scenarios presenting a wider variety of behavior change issues might need to be a key focus of future virtual patient developments in behavioral medicine.

This evaluation demonstrated the feasibility of integrating virtual patients into MOOCs in the health sciences and opens up the vista of future dissemination of counseling and treatment methods to students and practitioners in the health professions worldwide. In KIBEHMEDx, the percentage of MOOC participants obtaining a completion certificate was low at 3.8% of the original 19,000 enrollees. From a numerical perspective, 740 participants succeeded in completing 65% of course content and their responses suggest that some behavior change in an evidence-based direction occurred. Compared to in-classroom teaching with 35–40 students per term [23], the sheer number of students reached in one 5-week MOOC teaching period is equivalent to about 10 years of on-site instruction. An additional aspect to be considered is the cost of developing virtual patients; in this MOOC, the development of two interactive virtual patients required 150 h of work for the developers. The average development time needed to generate 1 h of interactive e-learning is 184 h [24], and given that course participants spent an average of 87 min to complete both virtual patient scenarios, the hypothetical effort expectancy would have been pegged at 267 h of development time [(184 h × 87 min)/60 min = 266.8 h (∼267 h)]. Clearly, the virtual patient development effort for this MOOC was relatively low in relation to the average cost of producing interactive e-learning.

From the point of view of the Best Evidence Medical Education approach to synthesizing evidence for effective educational interventions [25], however, it is not clear what level of impact the KIBEHMEDx MOOC can have had on in-clinic practices. Relying on self-reported performance measures in an environment without control over learners’ behavior can be unreliable. Dishonest behavior or surface learning in MOOCs is reported in literature [26] and was noticed in our course as well [11]. For this reason, MOOC certificates are not recognized by universities unless verified in a face-to-face setting or reliable biometric means such as webcam or keystroke dynamics profiles [27]. Ideally, learners would be aware that gaming in relation to the certificate granting mechanism results to cheating themselves; in fact, the edX platform has discontinued honor code-based certificates and now offers only fee-based verified certificates. Our purpose in including the virtual patient assignments was to contribute to knowledge development for those who understand how to participate in self-directed learning. Our evidence for such knowledge development is partly quantitative (e.g., numbers of participants, exit survey analysis, frequency/distribution of comments within the themes from the qualitative analysis). Our evidence for knowledge development and “clarification” (e.g., how the virtual patient intervention works, for whom and in what circumstances) is, on the other hand, to a large extent qualitative.

Another aspect we would like to emphasize is that the student target group for the KIBEHMEDx MOOC is a broad range of students in the health professions, practicing professionals as well as interested laypeople. The ambition to reach a varied scope of individuals is based on the idea that dissemination of knowledge about behavioral medicine can lead to improved health among laypeople as well as professionals. From a health sciences education perspective, our ambition has been one of inter-professional education, an integrated type of learning that is challenging to implement as well as evaluate [28]. We did not witness much interaction between different professions in the analyzed data, but we did not stimulate it explicitly in the tasks provided, which could be a point for improvement in upcoming editions of the MOOC. However, we encountered comments expressing gratitude for demonstrating the backstage of the therapeutic process. Many of the laypeople respondents declared willingness to apply the presented skills in relations with their relatives and friends. We do not see this as a danger but rather as an opportunity in this course, although unqualified self-treatment could be a potential issue in other healthcare-related MOOCs, an issue which should be openly discussed with a wider audience. Our approach has been to offer a virtual patient as a case-based method accessible to our broad target group. We see it as a strength that participants from multiple backgrounds were so overwhelmingly positive in terms of the helpfulness of virtual patient experience within the MOOC framework.

This evaluation has three principal limitations. First, our evaluation design did not include pre-course measurement, nor was any control group available for comparison. Secondly, the analysis is based on participant self-reported data, which has limitations in terms of trustworthiness of the answers and validity of self-assessment [29]. A third limitation was that we were unable to link data on student characteristics, entered on the edX platform, to data from learning trajectories in the virtual patient system. We were thus unable to report participant characteristics in relation to individual decisions made in the virtual patient. We hope that data-sharing agreements for analysis of student pathways through MOOCs will be in place as the development of this learning form rises exponentially.

### Future Research

As MOOCs develop further, future research should focus on objective evaluation of the virtual patients on knowledge, behavioral, and patient-related outcomes. One way of doing this would be to include pre-course measures of student knowledge and practice. Evaluating outcomes for patients treated via methods taught in MOOCs would be a significant challenge, however, for a MOOC like KIBEHMEDx. The generalist nature of the target group and the low learning commitment among most enrollees make this kind of evaluation nigh impossible. The content in a MOOC such as KIBEHMEDx can, however, be adapted to more narrowly defined target groups, like clinicians participating in Continuing Education (CE) in order to fulfill CE requirements or in order to develop their practice further. We would expect clinicians from a variety of health professions in a CE context to have a much higher course completion rate than the one achieved for KIBEHMEDx. Indeed, we see the online CE context as a major pathway for future dissemination and implementation of evidence-based knowledge in behavioral medicine, with potentially huge benefits for patients. Furthermore, the CE context would provide a better opportunity for more reliable evaluation of the impact of virtual patients on patient outcomes. Behavioral medicine CE MOOC-like modules could also be used by professionals working in an inter-professional, team-based context [30, 31] and could strengthen a shared inter-professional perspective on clinical practice goals [32]. The instructional format would also need to be re-designed to suit the work context of busy health professionals with limited time, perhaps in “micro-MOOCs.” Beyond the KIBEHMEDx focus on reducing the risk of noncommunicable diseases (NCDs) by controlling risk factors such as stress, sleep, and too little physical activity, CE MOOCs in behavioral medicine could be expanded to focus on techniques for managing chronic NCDs to attain maximum life quality. Evaluating patient outcomes would be a natural step to include in a future MOOC-based CE structure on behavioral medicine.

In conclusion, the great majority of course participants’ experience of the KIBEHMEDx virtual patients was highly positive. In the varied MOOC student body, consisting of health professionals, students, and laypeople, case-based learning seemed to work very well, indicating that virtual patients might well be introduced in other health-science related MOOCs. Future developments in behavioral medicine should be extended to continuing education (CE) for practicing health professionals; these could also facilitate team-based, inter-professional communication and practice in healthcare settings working collaboratively with the virtual patient scenarios in such CE MOOCs. This study focused on exploring participants’ perspectives on virtual patients in a health-related MOOC as well as on evaluating their satisfaction and self-reported attitude to behavior change outcomes. Future MOOC evaluations should collect objective data and answer to criteria regarding organizational practice and patient benefit in the form of patient outcomes.
